# The chronological change in transvaginal ultrasound images of a hemorrhagic ovarian cyst observed during infertility treatment: A case report and literature review

**DOI:** 10.1002/ccr3.4199

**Published:** 2021-07-09

**Authors:** Yu Fujii, Yu Wakimoto, Maya Omote, Yukiko Sugiyama, Yuji Ukita, Toru Kato, Atushi Fukui, Hiroaki Shibahara

**Affiliations:** ^1^ Department of Obstetrics and Gynecology Hyogo College of Medicine 1‐1 Mukogawa‐cho Nishinomiya Hyogo 663‐8501 Japan

**Keywords:** hemorrhagic ovarian cyst, ovarian tumor, transvaginal ultrasonography

## Abstract

Insights gained from chronological ultrasonogram images in the current case report will provide useful information for diagnosing and conservatively treating HOC. This could help avoid unnecessary laparotomy.

## Graphical abstract

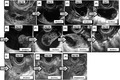

## 1 INTRODUCTION

Hemorrhagic ovarian cysts (HOCs) observed via ultrasound are not consistent over time and present different sonographic images depending on their size or echogenicity. Insights gained from chronological ultrasonogram images in the current case report will provide useful information for diagnosing and conservatively treating HOCs.

Treatment protocols for hemorrhagic ovarian cysts (HOCs) most often call for conservative management with clear guidelines set by the International Ovarian Tumor Analysis (IOTA). This case report discusses an atypical case and the outlines our reasons for deviating from a standard conservative treatment.

Here, we describe the successful treatment of a HOC in a 40‐year‐old patient who came to our hospital with the desire to conceive. However, there were a number of complicating factors that lead us to assume the presence of a malignant tumor instead of a HOC, which drove the decision to use a more aggressive treatment. Ultimately, a tumor was not found and our aggressive treatment exposed the patient to undue worry and unnecessary procedures.

Cases such as the one described in the report will likely become more frequent as women wait longer to conceive.^1^ This shift in the demographics of women will provide new challenges to medical care professionals as more women in the later stages of their fertility window seek fertility treatment. This report will hopefully act as a reference for future treatment and as further evidence for adhering to the guidelines set by the IOTA.

The IOTA recognizes that subjective assessment of HOC by an experienced ultrasound examiner is a preferred and widely accepted diagnostic measurement.^2^ As far as we know, only a few cases of HOC observed by ultrasonography change continuously with time from appearance to complete disappearance. We will outline our treatment process and why despite being an atypical case, conservative treatment is still recommended for similar cases.

## 2 CASE PRESENTATION

The patient was a 40‐year‐old, gravida 0 woman with a history of ureteral stones and was receiving treatment for Behcet's disease prior to entering our hospital. She expressed a desire to become pregnant to her primary physician and subsequently stopped receiving prednisone. A year prior, at 39 years old, the patient was diagnosed with multiple myoma by transvaginal ultrasound. Her primary physician referred the patient to our hospital for infertility treatment, which included a myomectomy followed by timed intercourse after monitoring her ovarian cycle via vaginal ultrasound. Laparoscopic‐assisted myomectomy was performed in our department, and infertility treatment was started 3 months postoperatively. Serum LH and FSH hormone levels were found to be within the normal range (Table 1). The patient indicated in her interview that she had regular menstrual cycles, but upon further investigation, this was found not to be the case. The results from the ultrasound monitoring showed an irregular cycle and that the patient met some of the criteria for PCOS, most notably a high AMH level.

**TABLE 1:** Hormonal date and Tumor marker

Hormonal date at first visit before myomectomy (day of measurement)	
LH(mIU/mL) (day 1)	5.6
FSH(mIU/mL) (day 1)	6.8
PRL(ng/mL) (day 1)	22.1
AMH(ng/mL) (day 1)	5.45
TSH(μIU/mL) (day 1)	1.9
FreeT3(pg/mL) (day 1)	3.13
FreeT4(ng/mL) (day 1)	1.17

Tumor marker	Cut‐off value
CA19‐9(U/mL)	14.2 (less than 37)
CA125(U/mL)	35.0 (less than 35)
HE4(pmol/L)	31.9
	(Premenopausal: less than 70)
	(Postmenopausal: less than 140)
ROMA (%)	0.024
	(Premenopausal <7.4%: low risk)
	(Postmenopausal <25.3%: low risk)

Note

The ROMA(risk of ovarian malignancy algorithm score) was calculated using the following Equation^13^

ROMA (%) = 100 × exp(PI) / [1 + exp(PI)]

Premenopausal: PI = −12.0 + 2.38 × LN(HE4) + 0.0626 × LN(CA125),

Postmenopausal: PI = −8.09 + 1.04 × LN(HE4) + 0.732 × LN(CA125),

exp(PI) = e^Pl^, LN = Log10.

**FIGURE 1:**
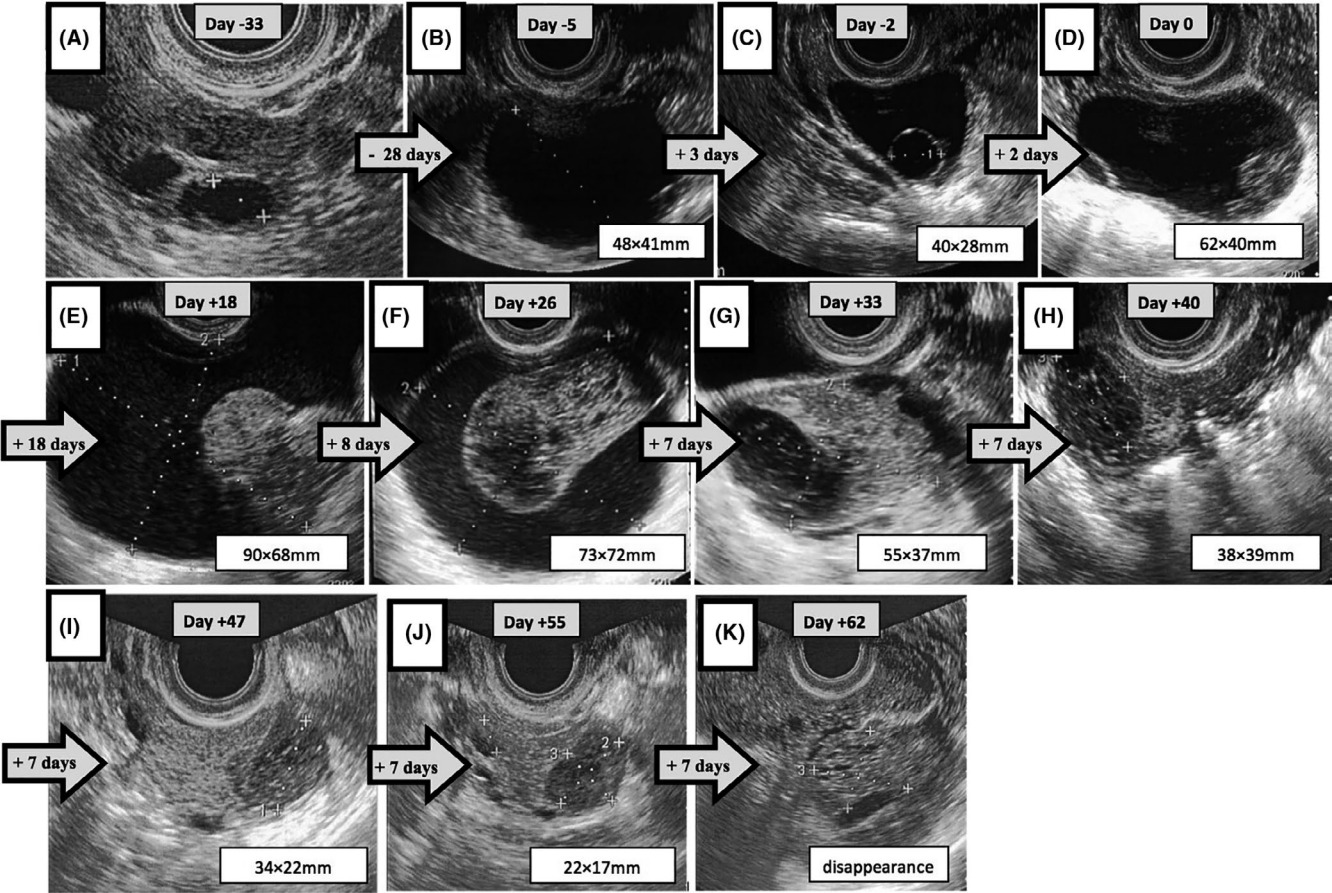
The chronological change in the transvaginal ultrasound images in hemorrhagic ovarian cysts. A, A normal right ovary with a small follicle on the 25th day of the menstrual cycle. B, An anechoic mass with a size of 48 × 41 mm on the 11th day of the menstrual cycle. C, An anechoic mass with a small follicle with a size of 40 × 28 mm on the 14th day of the menstrual cycle. D, A mass showing a solid pattern with a size of 62 × 40 mm on the 16th day of the menstrual cycle. E, A mass showing distinct solid parts with a size of 90 × 68 mm on the 6th day of the menstrual cycle. F, A mass showing a clear division into cystic and solid parts with a size of 73 × 72 mm on the 14th day of the next menstrual cycle. G, A mass mixed with dense echo and echo‐free spaces with a size of 55 × 37 mm on the 21st day of the menstrual cycle. H, A sponge‐like mass with a size of 38 × 39 mm on the 28th day of the menstrual cycle. I, A sponge‐like mass with a size of 34 × 22 mm on the 5th day of the menstrual cycle. J, A sponge‐like mass with a size of 22 × 17 mm on the 12th day of the next menstrual cycle. K, A normal right ovary after the mass disappeared on the 19th day of the next menstrual cycle. D indicates the first detection of HOC sonograms, which was defined as Day 0. A, B, and C are 33, 5, and 2 days before HOC was detected. D, E, F, G, H, I, J, and K are 18, 26, 33, 40, 47, 55, and 62 days after HOC was detected

Following standard treatment guidelines in Japan, the patient underwent timed intercourse with cyclofenil administered at a dose of 600 mg/d for 5 days, starting on cycle day 5 until cycle day 9. However, no follicular development was observed, even on the 25th day of the menstrual cycle. At this time, a mass was not observed in the right ovary on the ultrasonogram and a follicle width diameter of 8.6 mm was confirmed (Figure 1A). Starting on the 25th day of the menstrual cycle, 4 mg/d of chlormadinone acetate was administered for 10 days after which the patient experienced withdrawal bleeding. Following this, the patient underwent timed intercourse with clomiphene citrate, which was administered at a dose of 50 mg/day for 5 days starting on cycle day 5 until cycle day 9. On the 11th day of the menstrual cycle, a 48 × 41 mm anechoic mass was observed in the right ovary on the ultrasonogram (Figure 1B). We considered it a follicle and monitored its progress after obtaining informed consent from the patient. The mass changed to a 40 × 28 mm anechoic mass with a small follicle (Figure 1C). On the 16th day of the menstrual cycle, the mass size increased to 61 × 40 mm, showing a mixed pattern with a solid component (Figure 1D). We obtained a blood sample to identify tumor markers and serum E2 and P4 levels because the mass contained a solid component. Carbohydrate antigen 19‐9 (CA19‐9), carbohydrate antigen 125 (CA125), and human epididymis protein 4 (HE4) had normal values (Table 1). E2 and P4 levels were 595 pg/mL and 1.21 ng/mL, respectively. We judged that the mass was a follicle before ovulation. These results suggest that in Figure 1B the anechoic ovarian structure was a functional ovarian cyst or a persistent follicle. In Figure 1D, 5000 IU of human chorionic gonadotropin (hCG) was administered along with timed intercourse. On the 6th day of the next menstrual cycle, mass size increased to 90 × 68 mm, showing a mixed pattern with a solid component (Figure 1E). The patient had no symptoms. She received a followed up without any medication for infertility treatment. On the 14th day of the next menstrual cycle, the size slightly reduced to 73 × 72 mm with the solid part increasing in size (Figure 1F). Then, the mass became a 55 × 37 mm mixed mass with dense echo and echo‐free spaces on the 21st day of the menstrual cycle (Figure 1G). On the 28th day of the menstrual cycle, the mass changed to a 38 × 39 mm sponge‐like mass (Figure 1H). On the 5th day of the next menstrual cycle, it decreased to 34 × 22 mm (Figure 1I) and continued to gradually decrease in size (Figure 1J). At 62 days since detection (19th day of the menstrual cycle), the mass disappeared (Figure 1K), and we ultimately diagnosed the mass as a HOC according to its clinical course. Vaginal sonography was performed using a Mochida SONOVISTA FX, PE (7.5 MHz transvaginal probe) at an angle of 220° (Mochida Co.).

## 3 DISCUSSION

HOCs have variable sonographic appearances according to both hemorrhagic volume and time of hemorrhage occurrence.^3^ These echo patterns are due to images of blood flow or blood clotting in combination with images of precipitated fibrin.^4^ Immediately after bleeding, fresh blood is anechoic, progressing subacutely to a mixed echogenicity due to coagulation, and finally becoming anechoic due to hemolysis.^5^, ^6^ Thus, in our case, images in both Figure 1B and Figure 1C were considered to show an anechoic pattern due to the presence of fresh blood, a functional ovarian cyst, or a persistent follicle followed by the appearance of a mass separated into cystic and solid parts as the blood clot formed (Figure 1E,F).^4^ We were concerned about a potential rupture and the possibility of hemoperitoneum, so we continued to follow the developing mass until the size decreased. Using ultrasound, a solid part in the mass requires differentiation from malignant tumors. In the case of a HOC, the shape of the solid part is nearly straight and smooth and is not papillary when compared to that of a malignant tumor.^5^ Moreover, it is reported that the sonographic appearance of HOCs changes dramatically over a short period of time, and such characteristic changes have never been observed in the sonographic appearance of malignant tumors.^4^

We outlined some previous studies of HOC in Table 2. Several authors classified HOC echo patterns ranging between 3 and 7 identifiable types.^3^, ^4^, ^5^, ^6^, ^7^ In our case, the echo patterns could be roughly categorized as follows: anechoic cystic pattern, mixed pattern, mixed pattern with dense echo and echo‐free spaces, and sponge‐like pattern. The chronological sonographic images in our case followed the same course outlined by Okai et al, following type 1 to type 4 echo patterns. In our case, the mass in the right ovary showed an anechoic cystic pattern at the first detection (Figure 1B). If the bleeding was minimal and the blood was not completely coagulated, the internal echo of HOC could show a reticular pattern.^5^ Therefore, it is possible that the HOC at the first detection in our case (Figure 1B) was only follicular fluid. This interpretation is supported by the ultrasonogram taken after 3 days, as the mass size did not increase and the anechoic mass was maintained. Moreover, the high values of estradiol (almost 600pg/ml at the time of Figure 1D) confirmed this. We determined the cyst to be a follicle at that time. On the 11th day of the patient's menstrual cycle (shown in Figure 1B), anechoic ovarian structure presented as a functional ovarian cyst or a persistent follicle. This diagnosis is typically managed by induction with HCG or cycle canceling. At this time, the patient's hormonal level should have been checked, but the size of the follicle led to a differential diagnosis of a HOC. Had the patient's hormonal levels been checked, it would have given cause for a more conservative treatment from this point.

**TABLE 2:** The classification by transvaginal sonographic appearance of HOC and frequency of HOC sonogram type at first detection: literature review

References	Years of recruitment	Patients (n)	Age (years)	Echo pattern and the number of HOC cases											Size of HOC at the first examination	**The mean time interval for the mass disappearance**
Abbas AM et al^7^	11/2013 ~ 10/2014	48	28.1 (15‐50)	a diffuse dense echo pattern mimicking a solid mass 8 (16.7%)			a sponge‐like pattern 25 (52.1%)				a mixed cystic‐solid pattern 15 (31.2%)				4.8 cm	2‐6 wk
Okai T et al^5^	3/1989 ~ 2/1992	24	NA	a diffused echogenic pattern that seems to consist of a blood clot 5 (20.8%)		mixed pattern with a clearly demarcated solid part 9 (37.5%)			heteroechoic sponge‐like reticular pattern 3 (12.5%)				cysts including vague echo inside a cotton‐like pattern 7 (29.2%)		4.61 ± 0.97 cm (3.,3cm‐7.8 cm)	4.3 ± 2.0 wk (1‐8 wk)
Ding Z et al^3^	6/2002 ~ 6/2008	104	30 (13‐52)	a diffused dense echo pattern 21 (20.2%)		a mixed pattern 25 (24.0%)			a sponge‐like pattern 30 (28.8%)				a cystic pattern 28 (27.0%)		5.12 ± 1.33 cm (2cm‐7.6 cm)	3.5 ± 2.4 wk (X‐10weeks)
Nemoto Y et al^4^	NA	112	29.8 ± 8.2 (13‐52)	hyperechoic and hypoechoic solid type 38 (33.9%)		reticular or sponge‐like type 43 (38.4%)			mixture type of solid and cystic components 27 (24.1%)				cystic type 4 (3.6%)		4.48 ± 0.97 cm	4 wk (2‐8 wk)
Baltarowich OH et al^6^	NA	76	30 (17‐64)	Anechoic 0 (0%)	Homogeneous echoes: hypoechoic 8 (10.5%)	Homogeneous echoes: hyperechoic 5 (6.6%)		Heterogeneous echoes: predominantly anechoic with hypoechoic material 31 (40.8%)		Heterogeneous echoes: predominantly anechoic with hyperechoic material 12 (15.8%)		Heterogeneous echoes: predominantly hypoechoic 9 (11.8%)		Heterogeneous echoes: predominantly hyperechoic material 11 (14.5%)	5cm (2.5‐14 cm)	5.5 wk (1.5‐12 wk)

It is noteworthy that the patient had a regular menstrual cycle at 28 days, despite the ovarian structure. This suggests that it was not hormonally active and did not interfere with other follicle development. The HOC had a standard evolution, disappearing in 60 days. The high AMH value is unusual for the patient's age and is more commonly associated with polycystic ovaries in younger women. Taking into account the patient's diagnosis and cessation of treatment for Behcet's disease, her age, and high AMH value, a more aggressive approach was used to rule out the possibility of a malignant tumor.

In general, conservative management is recommended for HOC treatment.^7^, ^8^ However, a ruptured HOC can cause hemoperitoneum.^9^ If the patient has severe abdominal pain, increased white blood cell count, low hemoglobin level, or vital signs suggestive of shock, surgical management should be considered.^7^ Premenopausal patients suspected of having HOCs should be followed up for at least 2 months, provided that the patient has nonurgent conditions or clinical and laboratory data do not suggest a malignant tumor.^5^ Therefore, in our case, the patient's serum ovarian tumor marker levels of CA19‐9, CA125, and HE4 were analyzed to rule out malignant ovarian tumors.

Recently, HE4 has been reported to be a useful marker in ovarian cancer diagnosis.^10^ The risk of ovarian malignancy algorithm (ROMA) index, which is calculated by the risk prediction model for ovarian cancer using serum HE4 and CA125 levels, is more sensitive than HE4 alone and more specific than CA125 alone.^11^ We used tumor markers and the ROMA index to discriminate between benign and malignant ovarian tumors. It is important to note that Doppler was not available at the time of diagnosis, and as a result, vascularization was not able to be evaluated. According to the IOTA rules, the patient's case history does not require management with tumor markers.^2^ The IOTA rules are very effective in discriminating a benign structure in most cases. A study by Van Gorp et al showed that ultrasound methods were superior to ROMA when compared with the ability of the ROMA to diagnose ovarian cancer to that of greyscale and color Doppler ultrasound.^12^ Thus, such frequent monitoring and the use of the ROMA index score might not be necessary and may induce anxiety in the patient. We will review its use in our hospital and consider a more conservative treatment protocol.

In conclusion, the chronological ultrasonogram changes in the current case report provide useful information for managing HOC. Conservative treatment is still recommended in older patients presenting with irregular ovarian ultrasound images. This conclusion strengthens our stance that conservative treatment should always be considered in cases similar to the one described in this case report.

## CONFLICT OF INTEREST

None declared.

## AUTHOR CONTRIBUTIONS

YF and YW: wrote the first draft of the manuscript and contributed to finalization of the manuscript. MO, YS, and YU: assisted in the preparation of the manuscript. TK, AF, and HS: supported writing the manuscript and supervised the study. All authors: reviewed and approved the final manuscript.

## ETHICAL STATEMENT

This case report has been performed in accordance with the principles stated in the Declaration of Helsinki.

## Data Availability

All useful data are included in this manuscript.
